# Gut microbiota and sarcoidosis: a concise review

**DOI:** 10.3389/fmed.2026.1747012

**Published:** 2026-02-20

**Authors:** Roberto G. Carbone, Francesco Puppo, Victor F. Tapson

**Affiliations:** 1 Department of Internal Medicine, University of Genoa, Genoa, Italy; 2 Department of Medicine, Cedars-Sinai, Los Angeles, CA, United States

**Keywords:** autoimmunity, gut microbiome, infection, interstitial lung disease, lung microbiome, sarcoidosis

## Abstract

Microbial involvement in sarcoidosis pathogenesis is suggested by the observation that histological findings in sarcoid granulomas are like those of leprosy, tuberculosis and parasitic infection. Some studies have shown that the lung microbiome in patients with sarcoidosis is different from healthy individuals. Results are conflicting, reporting an abundance or decrease in bacterial and fungal species. The altered composition of the microbiome in sarcoidosis can contribute to the formation of granulomas, typical lesions of the disease, through interactions with the host immune system. However, no single microbe has been clearly demonstrated as a cause of sarcoidosis, several microorganisms have been involved in the formation of granulomas and are under study. In fact, various microorganisms have been detected in sarcoid granulomas and in the tissue of different organs. Microorganisms were demonstrated at the genomic level and only a few studies showed microbial presence using bacteriological or proteomic methods. A possible microbial involvement in sarcoidosis pathogenesis is further supported by studies reporting innate immune system activation and increased inflammatory cytokines secretion. Of note, a meta-analysis involving over 6,000 patients identified a strong association between *Cutibacterium acnes* and *Mycobacterium tuberculosis* and sarcoidosis. Interestingly, some studies have compared microbiomes in sarcoidosis with chronic respiratory conditions like chronic obstructive pulmonary disease, asthma, interstitial lung disease, and occupational lung diseases. Little is known whether gut microbiota alteration plays a causal role in the development of these diseases or is a consequence of a shared risk factor profile. However, current evidence does not conclusively support the causative role of microbes in sarcoidosis. Furthermore, research is studying the role of intestinal microbiomes in sarcoidosis with some studies showing that the restoration of the intestinal microbiome could be a possible therapeutic approach. The aims of the review are: (1) to clarify microbial involvement in sarcoidosis pathogenesis, (2) to describe microbiota in lungs of patients with sarcoidosis and to compare the data with other interstitial lung diseases.

## Introduction

Sarcoidosis, a disease characterized by noncaseating granulomas formation in various organs, is thought to involve interactions between the host immune system and microbiome, particularly in the lungs, gut, and blood (1–5). However, the exact origin of sarcoidosis is yet unknown.

Several studies report on the possible relationship of sarcoidosis with an autoimmune process while others suggest a possible close familial genetic correlation with sarcoidosis development. Furthermore, additional studies hypothesized that microorganisms such as *Cutibacterium acnes* and *Mycobacterium tuberculosis* could be involved in sarcoidosis development. Because of possible infectious mechanisms as a cause of disease, antibiotics have been included in the treatment approach. Current research suggests that changes in microbiota, particularly in the lungs, may play a role in sarcoidosis progression.

Interestingly, some sarcoidosis patients may have an increased risk of lymphoma, developing a condition defined as sarcoidosis-lymphoma syndrome, where sarcoidosis precedes lymphoma (6). Moreover, sarcoidosis or sarcoid-like granulomas have been reported in patients suffering from cancer, especially those treated with immunotherapy, perhaps related to excessive immune response against cancer cells (7–10).

The link between sarcoidosis and microbiome is supported by the hypothesis that changes in the microbiome community could influence its development especially in the lungs and gut. Research suggests a potential role for microbial dysbiosis (an imbalance in the microbiome) in immune dysregulation that drives sarcoidosis, possibly through the gut-lung axis where gut microbiota influence lung health. Specific microbial shifts have been observed in the airways and blood of sarcoidosis patients and some microorganisms like *Cutibacterium acnes* and *Mycobacterium tuberculosis* have been implicated.

The aims of the review are: (1) to clarify the role of microbial involvement in sarcoidosis pathogenesis, (2) to describe microbiota in lungs with sarcoidosis and to compare the data with other interstitial lung diseases.

## History of lung microbiome

The history of the lung microbiome began with the outdated belief that the lungs were sterile; a view disproven by new sequencing technologies in the 2010s. Early studies identified a bacterial community in the lower respiratory tract and subsequently linked the lung microbiome to diseases such as chronic obstructive pulmonary disease (COPD), cystic fibrosis, and pneumonia. This emerging topic is now exploring the roles of the entire microbiome including its interaction with the immune system and its connection to conditions such as lung cancer and COVID-19 infection.

The history can be divided into three sections: pre-2010, 2010–2019 and post −2019 to present. Around 2010, the sterile lung theory was dispelled based on the discovery of several bacteria in the lower respiratory airways even in healthy subjects. Thereafter, mycobiome (fungi) and viruses were included. During the 2014–2016 period the relationship between lung microbiome and pulmonary diseases began to be uncovered with studies regarding COPD, cystic fibrosis, pneumonia, and interstitial lung disease (ILD). The early study of lung sarcoidosis, a form of ILD associated with microbiome, has been reported by Garzoni et al. in 2013 (11). At that point, the role of the lung microbiome was linked to immune responses and this became a major focus. From the end of 2019 to the present, research has found significant connections between the lung microbiome, lung cancer, and COVID-19 infection (12).

## Sarcoidosis and microbiome

Many years ago, Du Bois et al. (13) proposed the possible microbial involvement in sarcoidosis pathogenesis based on the finding of numerous microorganisms in sarcoid granulomas. Interest in the gut microbiome’s role in sarcoidosis has grown in recent years, whereas the role of the pulmonary microbiome is largely unknown (14). Changes in the microbiome, particularly in the lungs, are believed to result in an immune response probably exacerbating the state of the disease in combination with pulmonary fungal and bacterial infections. A low number of antimicrobial peptides in the lung could lead to dysbiosis representing a pathogenic trigger for sarcoidosis development (14).

Many microorganisms have been identified at the genomic level, whereas only a few studies have demonstrated the presence of microbes using bacteriological or proteomic procedures. Notably, using monoclonal antibodies, *Cutibacterium acnes* was present in 63% of granulomas in cardiac sarcoidosis. These microorganisms introduce foreign substances, inducing an immune response. Moreover, mycobacteria produce mycobacterial heat shock proteins, superoxide dismutase A, and mycobacteria catalase-peroxidase and may be responsible for granuloma formation.

Lung sarcoidosis is associated with microbial dysbiosis, meaning an imbalance of bacteria and fungi in the lungs, which is linked to lower levels of antimicrobial peptides (AMPs) and an increase in microbes such as *Aspergillus*. Lower AMPs in sarcoidosis demonstrate an interaction between microbiota and immune system. The pathogenic role played by dysbiosis in sarcoidosis development needs to be confirmed by large experimental studies (14).

*Candida and Aspergillus* are the most common fungi detected in sarcoidosis, as confirmed by an increased level of antifungal antibodies in bronchoalveolar lavage (BAL) fluid and serum, suggesting that fungal infections may represent possible sarcoidosis etiologic agents (15).

Dysbiosis is an emerging area of research, and it is still being investigated whereas it is a cause or a consequence of sarcoidosis. Microbial/fungal imbalance may play a pathogenic role and could potentially be a target for future treatments. A higher abundance of *Aspergillus* has been observed in the lower airways of sarcoidosis patients. Additionally, increased levels of *Atopobium* and *Fusobacterium* were identified in sarcoidosis samples. Some research suggests that several fungal components, like those from *Aspergillus nidulans,* may trigger an immune response (CD + T cell activation) in sarcoidosis patients (16, 17).

## Gut and lung axis

The link between sarcoidosis and microbiome is supported by the hypothesis that changes in microbiome community could influence sarcoidosis development, especially in the lungs and gut. Research suggests a potential role for gut dysbiosis in the immune dysregulation that drives sarcoidosis, possibly through the gut-lung axis where gut microbiota may influence lung health. This concept implies that the gut microbiome communicates with the lungs. An unhealthy gut microbiome may trigger an exaggerated immune and inflammatory response in the lungs leading to sarcoidosis development. Therefore, changes in gut microbiome composition and function have been linked to inflammatory processes and granuloma formation in sarcoidosis (18).

Specific microbial shifts have been observed in the airways and blood of sarcoidosis patients and some microorganisms like *Cutibacterium acnes* and *Mycobacterium tuberculosis* have been implicated. Different bacterial and fungal components have been detected in the lower airways of sarcoidosis patients compared to healthy individuals, indicating a potential microbial imbalance. Furthermore, specific microbial genera have been identified that are more or less abundant in the blood of sarcoidosis patients and suggested that this finding could be a potential biomarker for the disease.

Pathways in amino acid and energy metabolism, redox process, and iron transport are significantly underrepresented in sarcoidosis patients. This suggests that microbial metabolites such as short-chain fatty acids which normally have immunomodulatory properties, may be altered and contribute to inflammation. Microbial antigens or their components can circulate through blood from the gut to other organs where they may trigger T-helper1 (Th1) and Th17 immune responses characteristic of granuloma formation (19). Specifically, activation and secretion of pro-inflammatory cytokines (IL-6, IL-12, IL-18, and TNF-alfa) contribute to alterations in gut microbiota and promote sarcoid granuloma production in various organs (19). Moreover, microbes are a source of ligands whose interplay with pattern recognition receptors of innate immunity cells may contribute to granulomatous inflammation (20).

**Figure 1:**
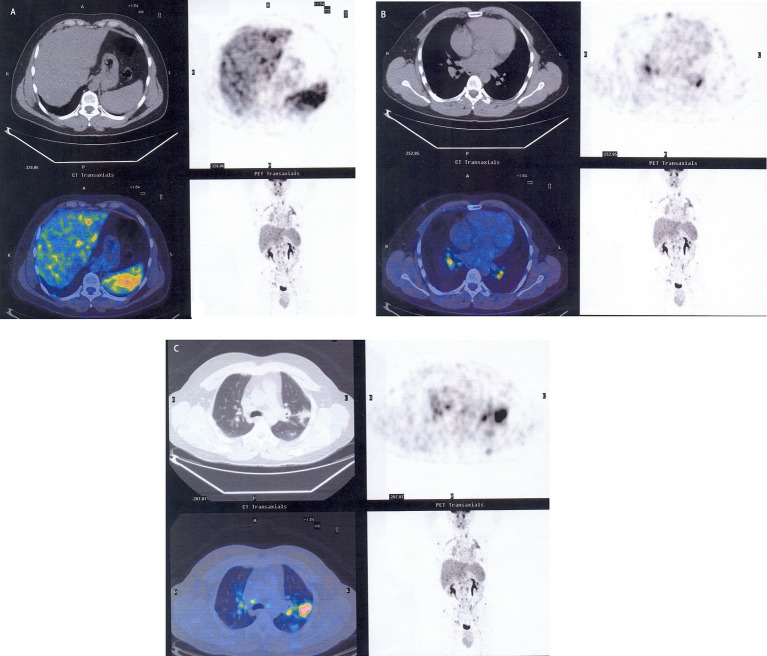
The figures highlight the sites affected by sarcoidosis through PET/CT scan: liver and spleen **(A)**, bilateral hilar lymph nodes **(B)**, subpleural area of the left upper lobe **(C)**.

**Figure 2:**
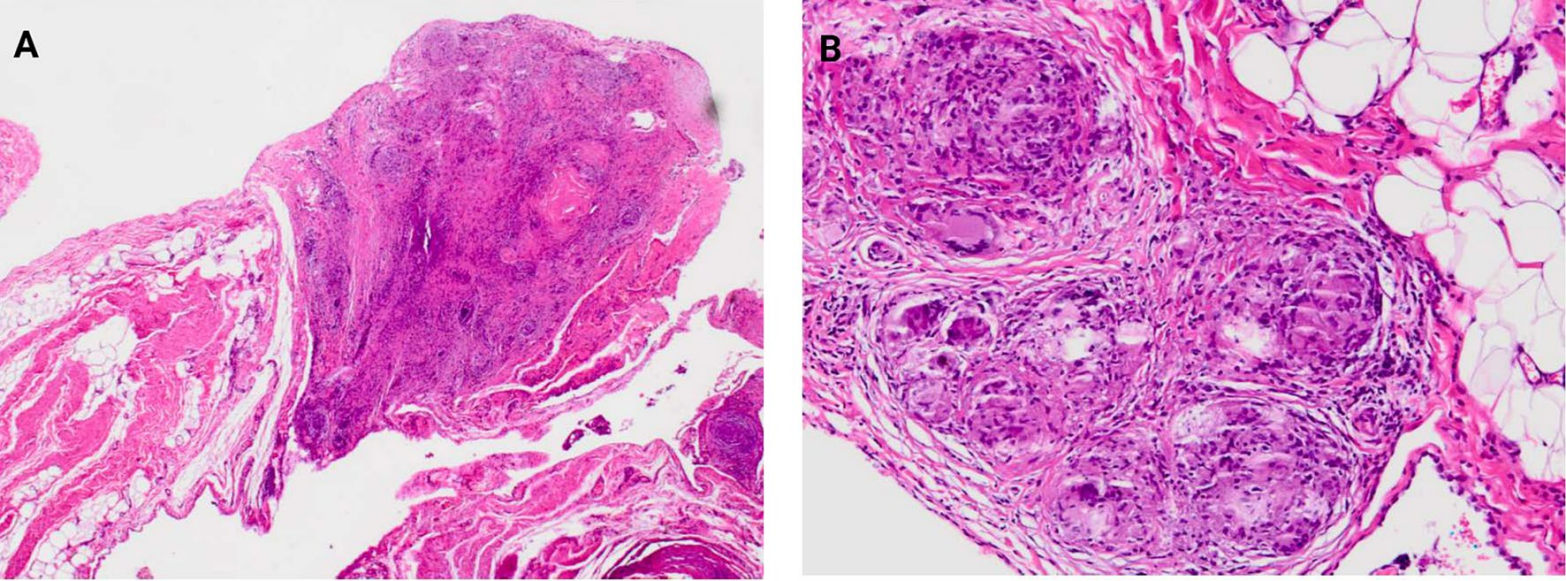
The figures highlight the omentum **(A)** with granulomas, H&E magnification X40 and at higher magnification sarcoidosis granulomas **(B)** in the same location, H&E magnification X100.

Sarcoidosis is usually diagnosed through a positron emission tomography/computed tomography (PET/CT) scan to exclude disease-affected lung and extrapulmonary sites. A definitive diagnosis of sarcoidosis is made through histology (Figures 1A–C, 2A,B) (21). Interestingly, increased levels of *Cereibacter sphaeroides* which are capable of heme synthesis, correlate with heme-related inflammation, a process potentially responsible for granuloma formation.

Finally, altered microbiome composition can affect gut integrity, potentially leading to increased intestinal permeability which allows microbes and their products to enter the systemic circulation.

## Genetics

Sarcoidosis development is closely influenced by genetic factors which interact with the microbiome. This includes: (i) genetic predisposition, (ii) key genes, (iii) and gene-microbiome interaction. The premise of genetic susceptibility is supported by familial clustering and a higher incidence of sarcoidosis within specific ethnic groups. Additionally, some genetic loci have been identified within human leukocyte antigens (HLA) region, as *HLA -DRB1, HLA-B,* and *BTNL2* that are linked with sarcoidosis development. Moreover, variants of other non-HLA genes, like *ANXA11* and *XAF1*, may increase granuloma duration (22).

Interestingly, specific HLA alleles associated with sarcoidosis, like *HLA-DRB 103*, have been linked to T cell responses to certain fungal antigens such as *Aspergillus nidulans*. Certain genes associated with sarcoidosis risk such as *SLC11A1* are also involved in susceptibility to certain bacterial infections (i.e., *Mycobacterium* species) suggesting a complex gene-microbe interaction. Genomic studies are currently underway (e.g., the GRADS study) to integrate microbiome and genomic data from patients to better understand disease mechanisms and identify potential biomarkers or treatments. Current research highlights sarcoidosis as a complex disorder where the genetic background of the host interacts with environmental triggers, specific microbial antigens and gut dysbiosis, that drive chronic, dysregulated immune response and granuloma production (21).

## Proteomics

Prior research indicates a significant relationship between microbiome and proteomics suggesting that dysbiosis influences a host’s immune response and protein expression contributing to sarcoidosis development and progression.

Proteomics helps identify proteins that can serve as biomarkers for sarcoidosis, including zinc finger protein 688 and mitochondrial ribosomal protein L43 which is especially linked to severe disease. Studies on alveolar macrophages found that protein alterations are related to key cellular functions such as Fcy-mediated phagocytosis and clathrin-mediated endocytosis.

Proteomics data when combined with other omics data, such as transcriptomics, helps identify biological pathways involved in sarcoidosis such as those related to neuro-inflammation and integrin signaling. Identifying these protein alterations provides insights into disease pathogenesis and can lead to the discovery of new therapeutic targets.

Therefore, there is evidence of a connection between microbiome composition, the host’s immune response to microbial proteins and sarcoidosis development (14).

## Microbiome and interstitial lung disease

Microbiome imbalance or dysbiosis in gut microbiome is associated with ILD (23). Notably, lung microbiome impacts ILD progression and studies have shown a role for lung dysbiosis in the pathogenesis of some ILD such as idiopathic pulmonary fibrosis (IPF) and sarcoidosis. As previously described, this correlation is largely explained by gut-lung axis, a bidirectional communication pathway between the gut and the immune system of distal organs like the lungs. Sarcoidosis patients exhibit significant differences in their gut’s microbiome composition compared to healthy individuals (different beta diversity). In sarcoidosis patients, the proportion of the *phyla Actinobacteria and Firmicutes* are expanded. Specific species such as *Cereibacter sphaeroides* are significantly more abundant. Other genera as *Veillonella, Prevotella, Cutibacterium,* and *Streptococcus* have been found to be enriched in studies of sarcoidosis patients. In healthy controls, the proportion of *Bacteroides* and *Verrucomicrobia* are lower. Functional pathways of gut microbiome are also changed (12). When comparing sarcoidosis with ILD, the abundance of *Haemophilus, Stenotrhophomonas* and *Enterobacter* is higher in ILD while the abundance of *Cutibacterium and Neisseria* is higher in sarcoidosis (24).

The microbiota difference between sarcoidosis and other ILD are reported in Table 1. Moreover, 16S RNA gene studies of the lung microbiome in sarcoidosis patients did not show the same changes that have been reported in other ILD (25).

**Table 1:** Comparison between sarcoidosis and other interstitial lung diseases.

Comparison group	Observation in sarcoidosis group	Observation in other ILD group
Sarcoidosis vs. ILD	No difference between groups.	No differences in most comparative studies.
Sarcoidosis vs. AE-ILD	⬆ Prevotella, Streptococcus, and Veillonella compared to AE-ILD.	No differences between AE-ILD and IPF
Sarcoidosis vs. ILD vs. COPD	⬇ Proteobacteria and Firmicutes compared to COPD/AE-COPD.	⬆ Proteobacteria in ILD compared to other groups; Klebsiella and ⬆ Staphylococcus at the genus level.
Sarcoidosis vs. healthy controls	⬇ Bacterial in lower airways; ⬆ Aspergillus	Not comparable in most studies. Presence of dysbiosis with respect to controls.

The table compares the microbiome in sarcoidosis, interstitial lung disease, and other diseases, focusing on specific genera where differences have been observed.

## Therapeutic implications

The strong association between gut and sarcoidosis suggests that gut microbiome may offer potential innovative therapeutic strategies for sarcoidosis. These potential approaches include: (1) probiotic supplementation, (2) dietary changes, (3) antimicrobial use, (4) fecal microbiota transplantation (FMT).

Probiotic supplementation allows the restoration of gastrointestinal health. Dietary changes influence microbial composition. Antimicrobial use is still under investigation. FMT shows encouraging results in other conditions, which may also be explored in future research. More research is needed to validate the effectiveness of these innovative therapies in sarcoidosis treatment (26).

Antibiotics are primarily experimental or used for their immunomodulatory properties, rather than to treat active infections, in sarcoidosis treatment. Standard treatment typically relies on corticosteroids like prednisone to reduce inflammation.

In this context, a study reported that 63% of granulomas in cardiac sarcoidosis patients tested positive for *Propionibacterium acnes*. Of interest, bacteria were also found in 63% of high-grade inflammatory lesions, suggesting they may act as a trigger for the disease’s characteristic immune response (27). Although not standard care, some studies explored the efficacy of antibiotic regimens in sarcoidosis. An exploratory trial showed that 63.2% of participants saw clinical improvements in King’s Sarcoidosis Questionnaire (KSQ) lung scores after azithromycin treatment for chronic cough (27). Moreover, the CLEAR Regimen, a combination of levofloxacin, ethambutol, azithromycin, and rifampin, has shown potential improvement of lung function and skin lesions in some clinical trials (28). Lastly, low-dose minocycline or doxycycline have been used to induce remission in cutaneous sarcoidosis (29). Therefore, antibiotics remain an experimental treatment for sarcoidosis.

## Discussion

Little is currently known about the correlation between the microbiome and sarcoidosis. There is a growing area of research that utilizes modern techniques including genetic analysis and proteomics aimed at the evaluation of possible linkages between gut and lung dysbiosis and sarcoidosis development.

For decades, scientists suspected that sarcoidosis was caused by an infectious agent, but no single pathogen was consistently identified. Recently, gut and blood microbiomes have been explored to see how they are related to sarcoidosis. These studies suggest that sarcoidosis and granuloma formation are likely determined by a combination of genetic predisposition with environmental triggers including active immune response to an unknown microbial trigger or a dysregulated microbiome.

However, the sarcoidosis microbiome compared to the microbiomes in other ILD has not been consistent across studies (23, 26).

The exact cause of sarcoidosis remains unknown, the microbiome is considered a significant contributing factor, and ongoing research is focused on dissecting the mechanism by which microbial imbalances drive the disease’s immune pathology. In addition, most studies conclude that there is no unique microbial signature that clearly distinguishes sarcoidosis from other ILD in stable condition. The pulmonary microbiota in both conditions appears to be similar, often dominated by bacteria commonly found in the oral/upper respiratory tract. Differences are more apparent when comparing either disease group to healthy controls (for example, lower diversity in the presence of disease) or when comparing stable disease to acute ILD exacerbation (21, 26, 30, 31).
